# Native Osseous CaP Biomineral Coating on a Biomimetic Multi-Spiked Connecting Scaffold Prototype for Cementless Resurfacing Arthroplasty Achieved by Combined Electrochemical Deposition

**DOI:** 10.3390/ma12233994

**Published:** 2019-12-02

**Authors:** Ryszard Uklejewski, Mariusz Winiecki, Piotr Krawczyk, Renata Tokłowicz

**Affiliations:** 1 Chair of Construction Materials and Biomaterials, Institute of Materials Engineering, Kazimierz Wielki University, Karola Chodkiewicza Street 30, 85-064 Bydgoszcz, Poland; winiecki@ukw.edu.pl; 2 Laboratory of Biomaterials and Peri-implant Bioprocesses Engineering, Department of Process Engineering, Institute of Technology and Chemical Engineering, Poznan University of Technology, Berdychowo 4, 60-965 Poznan, Poland; renata.toklowicz@doctorate.put.poznan.pl; 3 Department of Applied Electrochemistry, Institute of Chemistry and Technical Electrochemistry, Poznan University of Technology, Berdychowo 4, 60-965 Poznan, Poland; piotr.krawczyk@put.poznan.pl

**Keywords:** multi-spiked connecting scaffold (MSC-Scaffold), biomimetic scaffold, CaP biomineral coating, combined electrochemical deposition

## Abstract

The multi-spiked connecting scaffold (MSC-Scaffold) prototype with spikes mimicking the interdigitations of articular subchondral bone is an essential innovation in surgically initiated fixation of resurfacing arthroplasty (RA) endoprosthesis components. This paper aimed to present a determination of the suitable range of conditions for the calcium phosphate (CaP) potentiostatic electrochemical deposition (ECD_V=const_) on the MSC-Scaffold prototype spikes to achieve a biomineral coating with a native Ca/P ratio. The CaP ECD_V=const_ process on the MSC-Scaffold Ti4Al6V pre-prototypes was investigated for potential V_ECD_from −9 to −3 V, and followed by 48 h immersion in a simulated body fluid. An acid–alkaline pretreatment (AAT) was applied for a portion of the pre-prototypes. Scanning electron microscopy (SEM), energy dispersive X-ray spectroscopy (EDS) and X-ray diffraction (XRD) studies of deposited coatings together with coatings weight measurements were performed. Themost suitable V_ECD_ range, from −5.25 to −4.75 V, was determined as the native biomineral Ca/P ratio of coatings was achieved. AAT increases the weight of deposited coatings (44% for V_ECD_ = −5.25 V, 9% for V_ECD_ = −5.00 V and 15% for V_ECD_ = −4.75 V) and the coverage degree of the lateral spike surfaces (40% for V_ECD_ = −5.25 V, 14% for V_ECD_ = −5.00 V and 100% for V_ECD_ = −4.75 V). XRD confirmed that the multiphasic CaP coating containing crystalline octacalcium phosphate is produced on the lateral surface of the spikes of the MSC-Scaffold. ECD_V=const_ preceded by AAT prevents micro-cracks on the bone-contacting surfaces of the MSC-Scaffold prototype, increases its spikes’ lateral surface coverage, and results in the best modification effect at V_ECD_ = −5.00 V. To conclude, the biomimetic MSC-Scaffold prototype with desired biomineral coating of native Ca/P ratio was obtained for cementless RA endoprostheses.

## 1. Introduction

Resurfacing joint endoprostheses, e.g., total resurfacing hip arthroplasty (THRA) endoprostheses, are a bone-tissue-preserving option offered for relatively young and active patients with advanced osteoarthritis (OA). In contrast to traditional long-stem total hip replacement (THR) endoprostheses requiring surgical removal of the femoral head and neck, for the current generation of THRA endoprostheses, the femoral head is not removed, but is instead trimmed and capped with metal components fixed in subchondral bone with cement and a short stem placed in the femoral neck [1,2]. An essential innovation in the fixation technique for components of THRA endoprostheses in the periarticular trabecular bone—entirely cementless interfacing by means of the biomimetic multi-spiked connecting scaffold (MSC-Scaffold)—was designed, manufactured, structurally and geometrically functionalized, and tested in our previous research [3,4,5,6,7,8]. The concept of multi-spiked (needle-palisade) fixation of RA endoprostheses components in bone was invented by Rogala [9,10,11].

Unmodified metallic surfaces of joint endoprostheses components interacting with the bone have low osteoconductive and osseointegrative behavior; therefore, surface modifications are essential to enhance their biocompatibility and biological performance. Calcium phosphate (CaP) bioceramics are widely used in the field of bone regeneration, both in orthopaedics and in dentistry, due to their good biocompatibility, osteoconductivity, and osseointegrativity [12,13,14,15,16,17]. CaPs are of special importance since they are the most important inorganic constituents of hard tissues in vertebrates [12,13,14]. Coatings on orthopaedic or dental implant surface with a layer of CaP have also proven to be an effective approach in providing the base material with enhanced biocompatibility, osteoconductivity and osseointegrativity [15,16,17].

Synthetic CaP coatings can be prepared using a variety of processes. In general, the commonly used methods can be divided into two groups, physical deposition techniques and wet-chemical techniques [15,18]. Physical methods include plasma spraying [19], pulsed laser deposition [20], low-temperature high-speed collision [21], radio-frequency magnetron sputtering [22], gas detonation spraying [23] and ion implantation [24]. Chemical methods include chemical vapor deposition [25], biomimetic deposition [26,27], hydrothermal methods [28], sol–gel deposition [29] and electrochemical methods [27,30,31,32,33,34,35,36,37,38].

CaP coating deposition on flat substrates has been widely investigated, while CaP deposition on the bone-contacting surfaces of complex geometrical shapes, e.g., of porous implants or additively manufactured scaffolds, have only been studied relatively rarely and quite recently [39,40,41]. Most CaP deposition methods have a line-of-sight requirement, which greatly limits choices in coating irregular shapes [36]. Only a few methods can be applied for complex-shaped or porous materials and scaffolds. Therefore, for improving the osteoinductive and osseointegrative behavior of the bone-contacting surface of the MSC-Scaffold, electrochemical methods are preferred due to its shape complexity. The commonly used technologies for this purpose are electrophoretic deposition (EPD) and electrochemical deposition (ECD) [30].

The ECD process can be carried out at room temperature and allows for the CaP surface modification of complex-shaped Ti-alloy implants, resulting in a non-delaminating CaP coating of ca 1 μm thickness characterized by relatively high adhesive strength in comparison with the EPD process, where the hydroxyapatite (HA) coating is obtained from a suspension containing HA particles. Without applying thermal post-processing by subsequent sintering, the EPD deposited HA coating delaminates [21,24,30,31].

In the ECD process, CaP coatings are formed from an electrolyte containing calcium nitrate, Ca(NO_3_)_2_, and ammonium dihydrogen phosphate, NH_4_H_2_PO_4_, wherein the weight ratio of calcium to phosphorus is ca. 1.67 and is the same as the ratio of Ca/P in the native osseous CaPs [32,42,43,44,45,46]. This method enables control of the properties of the deposited coatings by appropriately choosing the electric parameters of the ECD process, such as current density [47] or electric potential [29], and/or by adjusting the process time [23]. The subsequent immersion of the modified substrates in simulated body fluid (SBF) leads to the transformation of the amorphous CaP coating into a crystalline CaP coating [29,31]. Application of chemical pretreatment, like acid, alkaline or acid–alkaline treatment (AAT), may advantageously influence the outcome of the ECD process [48,49,50,51,52,53].

Attempts to modify the bone-contacting surfaces of the MSC-Scaffold pre-prototype by ECD of CaPs has been undertaken initially at constant current densities with subsequent immersion in SBF (to transform the amorphous CaP coating into bone-like biomineral coating) [54]. It was observed that the deposition of CaPs on surfaces of the MSC-Scaffold pre-prototypes can be controlled by adjusting the current density. These modifications were successfully performed in the galvanostatic ECD_j=const_ process, but continued research using the potentiostatic process (ECD_V=const_) showed significantly higher repeatability than the galvanostatic ECD process [55]. Similarly, the poorly investigated CaP deposition on complex-shaped substrates (e.g., scaffolds) and the unsatisfactory attempts during our preliminary research in applying ECD process parameters recommended for flat substrates to the MSC-Scaffold pre-prototypes, strongly justify the need to take an experimental approach for finding the suitable range of conditions for the CaP ECD_V=const_ process on the MSC-Scaffold pre-prototypes.

The particular aim of this paper was to present the determination of the suitable range of conditions for potentiostatic electrochemical deposition of calcium phosphates (CaPs) on the MSC-Scaffold prototypes to achieve a native biomineral Ca/P ratio in the coating, which is of great importance for good biocompatibility and biological performance of the implant in vivo. Good biological performance of this implant was proved in our recent investigation in swines on a partial knee arthroplasty (RKA) endoprosthesis working prototype with the CaP coated in potentiostatic ECD process MSC-Scaffold [56].

The main aim of our work is to elaborate on the suitable MSC-Scaffold prototypes for a new generation of entirely cementless RA endoprostheses.

## 2. Materials and Methods

### 2.1. MSC-Scaffold Pre-Prototypes

**Figure 1:**
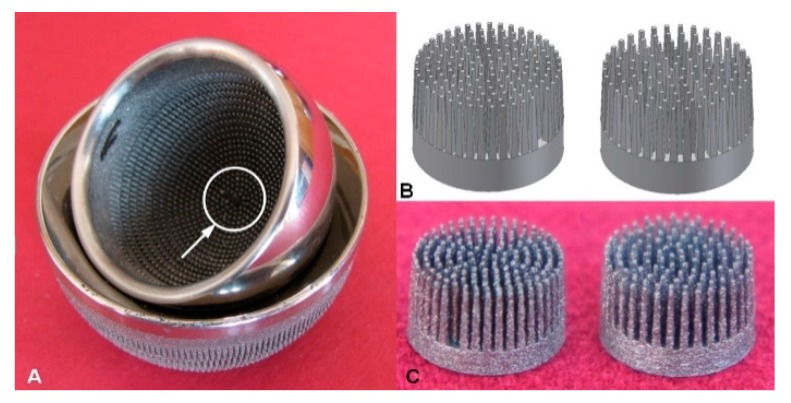
(**A**) Prototype of the entirely cementless total resurfacing hip arthroplasty (TRHA) endoprosthesis with the multi-spiked connecting scaffold (MSC-Scaffold) manufactured using selective laser melting (SLM) of Ti4Al6V powder; (**B**) CAD models of the MSC-Scaffold pre-prototypes for RHA endoprostheses designed in two geometrical configuration variants, which vary by the distance between the spike bases, 200 μm (P_Sc200_) and 350 μm (P_Sc350_), both circumferentially and radially, and (**C**) the MSC-Scaffold pre-prototypes manufactured on the basis of these CAD models using SLM.

Surface modification of the MSC-Scaffold prototype for non-cemented resurfacing joint endoprostheses was carried out on the MSC-Scaffold pre-prototypes designed as fragments of the central part of the femoral component of the TRHA endoprosthesis. The multilateral spikes of the MSC-Scaffold pre-prototypes were arranged in concentric parallel rings around the central spike with axes parallel to each other, whereas the central spike was coincident with the femoral head axis of symmetry. The length of the square side in the spike pyramid’s base was 0.5 mm in the MSC-Scaffold CAD model. The prototype THRA endoprosthesis with the MSC-Scaffold manufactured using selective laser melting (SLM) of Ti4Al6V powder is presented in Figure 1A. In Figure 1B, CAD models of the MSC-Scaffold pre-prototypes designed for this research are presented. The two design variants vary by the distance between spike bases, 200 μm (P_Sc200_) and 350 μm (P_Sc350_), both circumferentially and radially, which corresponds to the thickness of bone trabeculae of cancellous bone. In Figure 1C, the MSC-Scaffold pre-prototypes manufactured using SLM are shown. The manufacturing was subcontracted to the Centre of New Materials and Technologies at the West Pomeranian University of Technology in Szczecin, Poland. The process parameters applied during the SLM manufacturing were: laser power 100 W, layer thickness 30 µm, laser spot size 0.2 mm, scan speed 0.4 m/s and laser energy density 70 J/mm^3^.

### 2.2. Preparation of the MSC-Scaffold Pre-Prototypes’ Surfaces

After SLM manufacturing, to remove the adhered powder aggregates from the spike surfaces, a manual blasting treatment was carried out using an experimentally customized abrasive mixture composed of equal proportions of white aloxiteF220 (~53–75 μm), white aloxite F320 (~29.2 μm ± 1.5%), and blasting micro glass beads (~30 μm ± 10%) [57]. Cleaning in an ultrasonic bath (Sonic 3, Polsonic, Poland) was applied using the following agents distilled water, ethanol, acetone and, again, distilled water three more times; each stage of cleaning was carried out for 15 min. After that, the MSC-Scaffold pre-prototypes were dried at room temperature and the initial weight was measured using a precise analytical balance (AS 110/X, Radwag, Poland).

### 2.3. Determination of the Most Suitable Range of Conditions for the ECD_V=const_Process

To determine the most suitable range of potential (V_ECD_) for the ECD_V=const_ process, a total of 56 MSC-Scaffold pre-prototypes (28 of each variant) were subjected to surface modification. To search for the appropriate conditions of the ECD_V=const_ process, V_ECD_ values from −9 to −3V were investigated using a potentiostat-galvanostat apparatus (PGSTAT 302N, Metrohm Autolab, Ultrecht, The Netherlands). The CaP ions were deposited from a solution composed of 0.042 M calcium nitrate, Ca(NO_3_)_2_, and 0.025 M ammonium dihydrogen phosphate, NH_4_H_2_PO_4_, with pH=6. The ECD process was performed in a two-electrode system. The process was carried out for one hour at room temperature. A gold plate anode was used as the counter electrode. After the ECD_V=const_ process, the MSC-Scaffold pre-prototypes, playing the role of working electrode, were rinsed with distilled water and, to convert the deposited amorphous CaP coating into the bone-like biomineral coating, they were immersed for 48 h in an SBF solution composed of 6.8 g/L NaCl, 0.4 g/L KCl, 0.2 g/L CaCl_2_, 0.2048 g/L MgSO_4_∙7H_2_O, 0.1438 g/L NaH_2_PO_4_∙H_2_O and 1.0 g/L NaHCO_3_ at 37 °C. After the incubation in SBF, the MSC-Scaffold pre-prototypes were dried at room temperature and their final weight was measured. The weight increase due to surface coatings deposited on the spikes of the MSC-Scaffold pre-prototypes was calculated as the difference between the initial and final weights of the modified MSC-Scaffold pre-prototype. An analysis of chemical composition of the coating deposited on the lateral spike surfaces of the MSC-Scaffold pre-prototypes was performed using a scanning electron microscope (Hitachi TM-3030, Hitachi High-Tech Technologies Europe GmbH, Krefeld, Germany) equipped with the energy dispersive X-Ray (EDS) system (Oxford Instruments, Abingdon, UK). The results can be used for calculating the Ca/P ratios.

### 2.4. The Influence of the AAT Pretreatment

The same ECD_V=const_ surface modification process was performed to examine the influence of the AAT pretreatment on the final surface modification. In this step, the V_ECD_ values that provided the highest weight increase simultaneous with Ca and P contents with Ca/P ratios corresponding to the Ca/P ratios of native osseous CaP there were applied to new pre-prototypes. A total number of 36 MSC-Scaffold pre-prototypes were modified, 12 for each V_ECD_ value identified as favorable; half of the pre-prototypes underwent AAT pretreatment. The AAT process was conducted in 40% H_2_SO_4_ for 40 min at 60 °C and subsequently in 1 mol/L NaOH for 40 min at 80 °C.

EDS surface mapping of three randomly selected subareas of the lateral spike surfaces of each MSC-Scaffold pre-prototype was performed using a specialized software analyser in the EDS system used. Based on mapping analysis, the regions with CaP deposited on the spikes’ lateral surface were indicated and the coverage degree of the lateral spike surfaces was determined. In each of the analysed subareas, 10 pointwise measurements of the chemical composition were made, and the Ca/P ratios were calculated. The analyses of the coverage degree of the lateral surface of spikes and the deposited coating uniformity were made using the professional software tool ImageJ (National Institutes of Health, Bethesda, Maryland, USA). Structure and phase composition of the deposited coating was identified by XRD on a PANalytical EMPYREAN X-ray diffractometer (Malvern, UK) at a scanning speed of 0.02°/s with Cu Kα radiation (λ = 0.15405 nm, 40 mA, 40 kV) at a 2θ range of 30–70°. Since, there was no technical possibility to analyze the surface of MSC-Scaffold pre-prototypes directly, so to obtain the XRD roentgenograms we had to use the deposits detached mechanically from the spikes as a powder sample.

## 3. Results

### 3.1. Determination of the Most Suitable Range of Conditions for the ECD_V=const_Process

**Figure 2:**
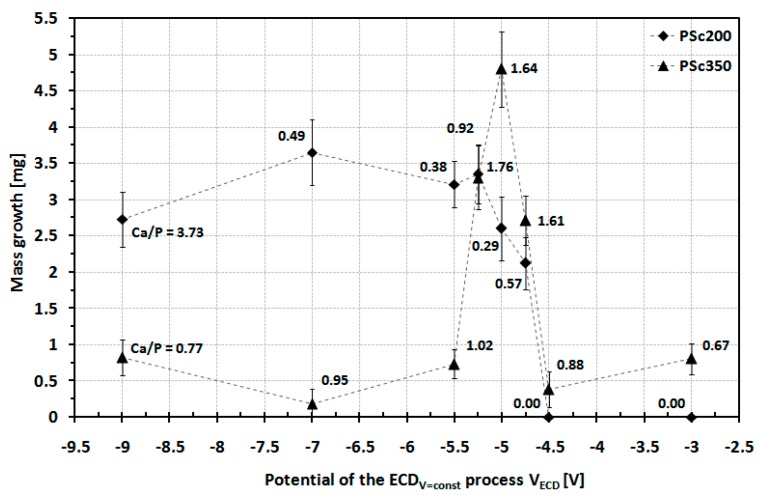
Mass increase of surface coating of the P_Sc200_ and P_Sc350_MSC-Scaffold pre-prototypes after the ECD_V=const_ process as a function of the applied V_ECD_ value.

Figure 2 shows a diagram of the mass increase due to the surface coating for the P_Sc200_ and the P_Sc350_MSC-Scaffold pre-prototypes as a function of the applied V_ECD_ values during the ECD_V=const_ process of CaP deposition.

**Figure 3:**
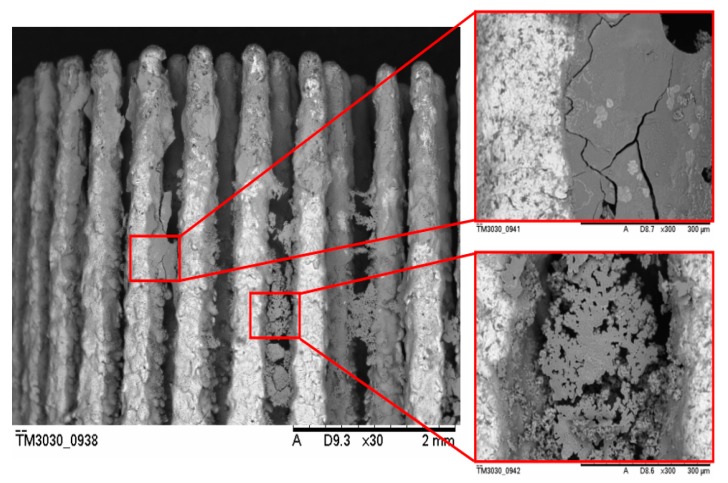
Example SEM images showing the unwanted effect of CaP deposition in the inter-spike space of the P_Sc200_ MSC-Scaffold pre-prototypes during the ECD_V=const_ process; magnification: 30× and 300×.

For the P_Sc200_MSC-Scaffold pre-prototypes modified using V_ECD_ values ranging from −9 to −5.25 V, the surface weight increase was about 3 mg for the initial stages (from 2.75 mg for V_ECD_ = −9 V to 3.65 mg for V_ECD_ = −7 V). Increasing the V_ECD_ value past −5.25 V resulted in reducing the deposited coating weight increase to 2 mg (for V_ECD_ = −4.75 V), while for V_ECD_ = −4.5 V and V_ECD_ = −3 V there was no noted weight increase. EDS analysis of chemical composition confirmed the absence of Ca and P on the lateral spike surfaces of the MSC-Scaffold pre-prototypes modified by applying V_ECD_ values of −4.5 V and −3 V. SEM analysis revealed that, for the P_Sc200_ MSC-Scaffold pre-prototypes for which a weight increase was observed, CaPs were deposited only on the upper regions of spikes. In this case, a significant amount of CaP deposit was found in the inter-spike space of the MSC-Scaffold pre-prototypes. This phenomenon was judged to be disadvantageous. Example SEM photographs showing this effect are presented in Figure 3. None of the Ca/P ratios determined for the lateral spike surfaces of the P_Sc200_ MSC-Scaffold pre-prototypes corresponds to the Ca/P ratio characteristic for CaPs. EDS analysis shows that the Ca/P ratios reached values below 1.00 and 3.73, so in this case, there was no CaP coating on the spike surfaces, but only Ca and P ions randomly deposited onto the surface of the MSC-Scaffold pre-prototypes’ spikes. In the first case, almost the entire surface was deposited with Ca whereas in the second case, nearly all deposits were P. At −4.5 V no mass increase was observed. 

**Figure 4:**
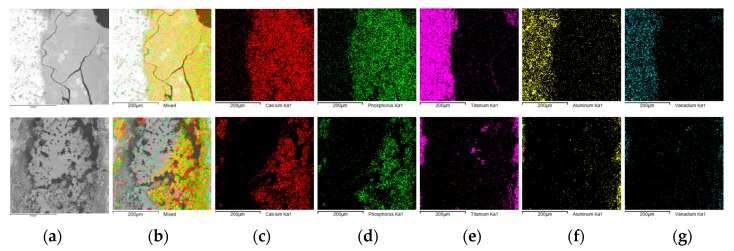
SEM and EDS mapping of the elemental species on the surface of the MSC-Scaffold pre-prototypes’ spikes and deposits between the spikes (**a**) SEM morphology, (**b**) CaP map, (**c**) Ca map, (**d**) P map, (**e**) Ti map, (**f**) Al map and (**g**) V map.

An example of the EDS chemical mapping of two magnified areas is presented in Figure 4 in which colors represent individual elements. As is clearly seen, the elemental species coming from the pre-prototype material, like Ti, V, and Al, are located on the lateral surface of the spikes (as is O, which is not shown) while Ca and P are distributed only as deposits in the inter-spike space. This phenomenon could be explained by considering the distance between the spikes. It is most likely caused by insufficient room between the spikes. The unwanted result of the CaP ECD_V=const_ surface modification of the P_Sc200_ MSC-Scaffold pre-prototypes led to the decision to abandon further research using this geometrical variant of MSC-Scaffold pre-prototype.

For the P_Sc350_ MSC-Scaffold pre-prototypes modified using V_ECD_ values ranging between −9 to −5.50 V, the weight increase of the surface coating was low (less than 1 mg) and the Ca/P ratios determined in the deposited surface coatings did not correspond to the Ca/P values characteristic for CaPs. A significant increase in weight (about 5 mg) was found when applying V_ECD_values ranging between −5.25 to −4.75V. For the ECD process carried out using V_ECD_ values above −4.50 V, a slight weight increase was observed (approximately 0.50–0.75 mg). Unfortunately, the Ca/P ratio in the deposited surface coating did not correspond to the characteristic Ca/P values for native osseous CaPs. EDS analysis of all the P_Sc350_ MSC-Scaffold pre-prototypes modified using V_ECD_ values of −5.25, −5.00 and −4.75 V confirmed the presence of CaPs having the Ca/P ratios consistent with the native osseous CaPs). Therefore, V_ECD_ values from −5.25 to −4.75V can be recommended as the most suitable conditions for the CaP ECD_V=const_surface modification of the P_Sc350_ MSC-Scaffold pre-prototypes.

### 3.2. The Influence of the AAT Pretreatment

**Figure 5:**
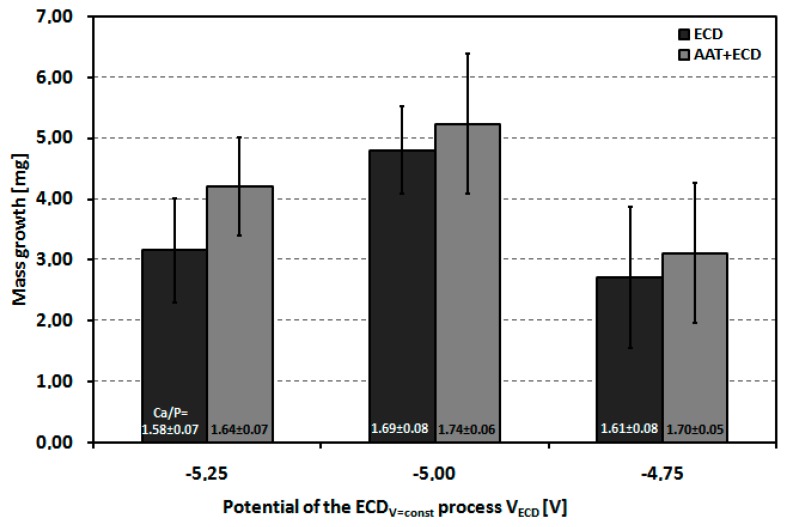
The average weight increase of the P_Sc350_ MSC-Scaffold pre-prototypes as a function of applied V_ECD_ values for the MSC-Scaffold pre-prototypes with and without AAT pretreatment.

Figure 5 shows the dependence of the average weight increase of the P_Sc350_ MSC-Scaffold pre-prototypes modified using the V_ECD_ values of −5.25, −5.00 and −4.75 V. The dependence was determined both for the MSC-Scaffold pre-prototypes that underwent the AAT pretreatment and those that did not.

In both cases, the highest average weight increase for the modified P_Sc350_ MSC-Scaffold pre-prototypes was obtained for V_ECD_ = −5.00 V. It can be clearly seen from Figure 4 that AAT pretreatment impacts the weight increase of the deposited CaP coating (by 44% for V_ECD_ = −5.25 V, by 9% for V_ECD_ = −5.00 V and by 15% for V_ECD_ = −4.75 V). 

**Figure 6:**
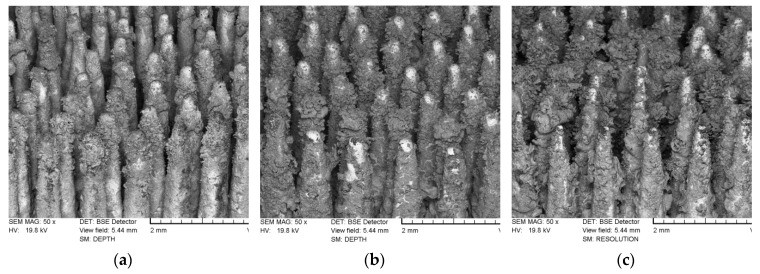

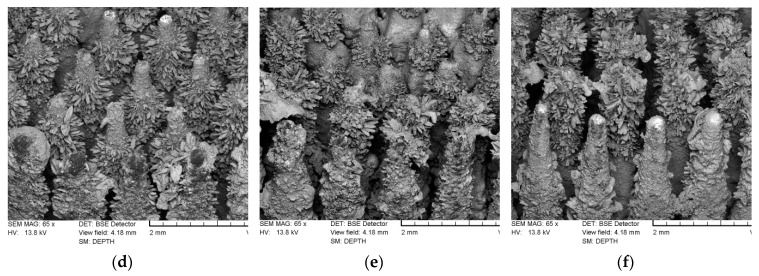
SEM images of the lateral spike surfaces of the MSC-Scaffold pre-prototypes modified by a one hour ECD_V=const_ process carried out using V_ECD_ values of: (**a**) −5.25 V, (**b**) −5.00 V and (**c**) −4.75 V, followed by 48 h incubation in SBF without AAT pretreatment, and, correspondingly, (**d**–**f**) with AAT pretreatment.

Figure 6 shows P_Sc350_SEM images of the lateral spike surfaces of the MSC-Scaffold pre-prototypes modified by a one hour ECD_V=const_ process carried out using V_ECD_ values of −5.25, −5.00, and −4.75 V, followed by 48 h incubation in SBF, without AAT pretreatment (Figure 6a–c) and with AAT pretreatment (Figure 6d–f).

SEM analysis of the microstructure of the lateral spike surfaces shows that the CaP coating obtained during the ECD_V=const_ process carried out without AAT pretreatment is non-uniform and seems to be unstable (the surface is not consistent). For MSC-Scaffold pre-prototypes modified using −5.25 V, most of the lateral spike surfaces remained uncoated in their medial part. The coating was deposited mostly on the upper part of the spikes. For the remaining pre-prototypes (modified using the V_ECD_ values of −5.00 and −4.75 V) the entire lateral surface of spikes was CaP coated, but numerous micro-cracks, especially for V_ECD_ = −5.00 V, were noted. 

As can be clearly seen in the SEM images presented in Figure 6d–f, applying an AAT pretreatment has increased the coverage degree of the spike surfaces and the uniformity (no micro-cracks appear on the spike surfaces) of the produced CaP coatings for all V_ECD_ values of the ECD_V=const_ process. Plate-like and needle-like shaped CaP crystals appear on the lateral surfaces of the MSC-Scaffold pre-prototypes. In particular, a significant accumulation of such crystals can be observed in the upper part of the MSC-Scaffold’s spikes. 

**Figure 7:**
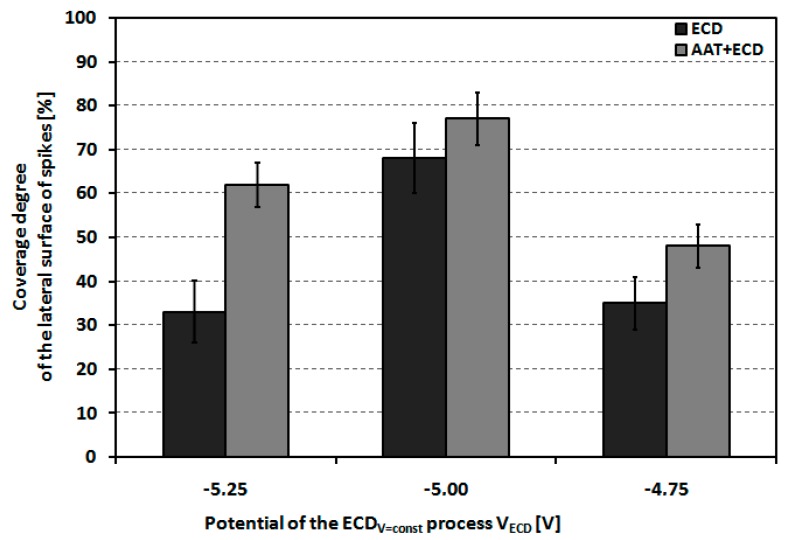
The coverage degree of the lateral spike surfaces of the P_Sc350_ MSC-Scaffold pre-prototypes after one hour E_CDV=const_ carried out at V_ECD_ values of −5.25, −5.00 and −4.75 V followed by 48 h incubation in SBF, with and without AAT pretreatment as a function of the applied V_ECD_.

**Figure 8:**
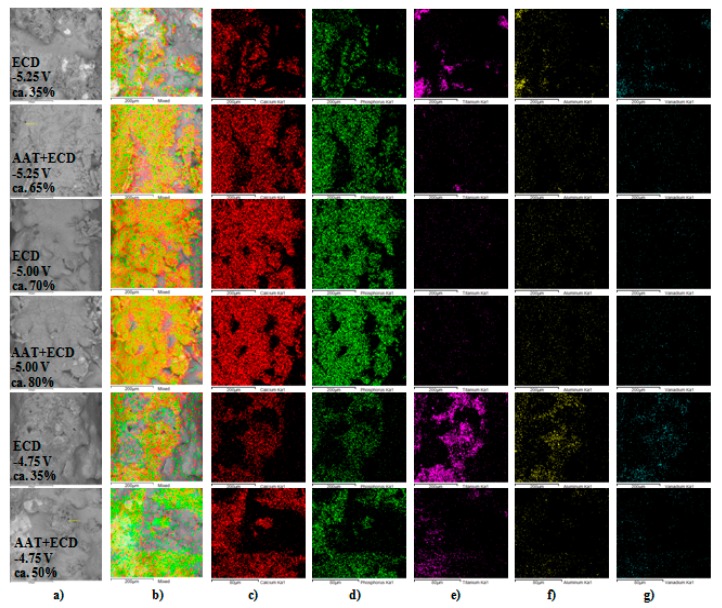
Example results of the EDS mapping of the elemental species on the surface of the MSC-Scaffold pre-prototypes’ spikes and deposits between the spikes: (**a**) SEM morphology, (**b**) CaP map, (**c**) Ca map, (**d**) P map, (**e**) Ti map, (**f**) Al map and (**g**) V map.

From the EDS analysis, the molar ratios of calcium to phosphorous on the lateral spike surfaces were 1.58–1.74, which is consistent with the values of native osseous CaP. The graph in Figure 7 shows the coverage degree of lateral spike surfaces of P_Sc350_ MSC-Scaffold pre-prototypes that underwent one hour of ECD_V=const_ process carried out using V_ECD_ values of −5.25, −5.00 and −4.75 V followed by 48 h immersion SBF, with and without AAT pretreatment as a function of the applied V_ECD_ during the ECD_V=const_ process. Figure 8 shows examples of the EDS chemical mapping for the lateral spike surfaces of the MSC-Scaffold pre-prototypes. The examples correspond with the results presented in Figure 7.

The EDS mapping results for the modified lateral spike surfaces and the quantitative analysis performed using ImageJ show that the greatest lateral spike surface coverage degree was obtained for the P_Sc_350 MSC-Scaffold pre-prototypes modified at V_ECD_ = −5.00 V (average 68±6%). For other V_ECD_ values, the coverage degree of the lateral spike surfaces was half the size (33±5–35±5%). Applying of AAT pretreatment increases the coverage degree of the lateral spike surfaces (40% for V_ECD_ = −5.25 V, 14% for V_ECD_ = −5.00 V, and 100% for V_ECD_ = −4.75 V).

### 3.3. XRD Analysis

**Figure 9:**
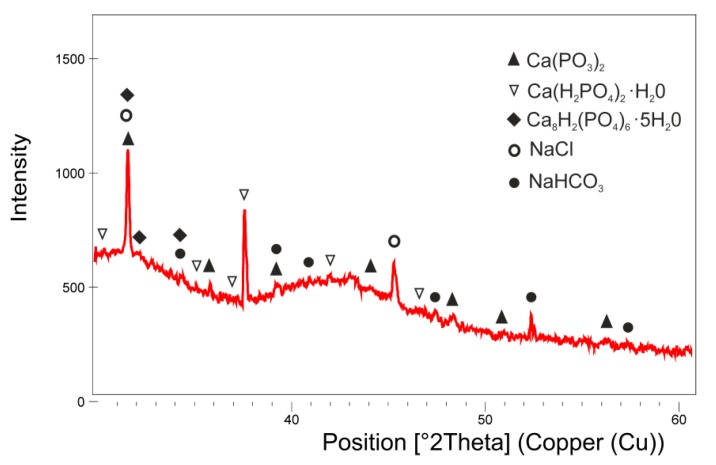
XRD pattern of the MSC-Scaffold pre-prototype coated with a layer of CaP deposited by ECD_V=const_ at V_ECD_ = −5.00 V (followed by 48 h immersion in SBF).

To confirm the crystalline form of CaP was deposited on the surface of MSC-Scaffold pre-prototypes due to electrochemical treatment using V_ECD_ = −5.00 V, an XRD analysis was performed. The results are shown in Figure 9.

As one can see, the obtained CaP coating is multiphasic and there are identified peaks attributed to CaP phases like octacalcium phosphate, Ca_8_H_2_(PO_4_)_6_∙5H_2_O (PDF2# 00-026-1056), calcium metaphosphate, Ca(PO_3_)_2_ (PDF2# 00-003-0348), and monocalcium phosphate monohydrate, Ca(H_2_PO_4_)_2_∙H_2_O (PDF2# 00-003-0284). Apart from this, the impurity phases from the SBF solution were found on the surface sodium chloride, NaCl (PDF2# 01-077-2064), and sodium hydrogen carbonate, NaHCO_3_ (PDF2# 00-021-119). The results of the XRD investigation showed that, during the combined ECD process conducted under the determined conditions, CaP biomineral coating is produced on the lateral surface of spikes of the MSC-Scaffold.

## 4. Discussion 

In this study, the experimental CaP modification of the bone-contacting surfaces of MSC-Scaffold pre-prototypes was undertaken in search of the suitable range of conditions for CaP deposition in the ECD_V=const_process. Since the MSC-Scaffold prototype, as the original concept of a multi-spiked (needle-palisade) fixation of RA endoprostheses components, was developed within the frames of two of our research projects, its most suitable geometrical properties evolved based on the findings of bioengineering research in relation to that primarily suggested in the patented version [3,4,5]. Additionally, the conditions ECD modifications have changed accordingly. Attempts to modify the bone-contacting surfaces of MSC-Scaffold prototypes initially undertaken at constant current densities were satisfactory [52], i.e., it is possible to control the deposition of CaPs on the bone-contacting surfaces of MSC-Scaffold pre-prototypes by adjusting the current density [52].Significant enhancement of the osteoinduction/osseointegration potential of the MSC-Scaffold prototype was confirmed in pilot experimental studies in animal models and in osteoblast cultures [53]. After the structural-geometric functionalization of the additively manufactured prototype MSC-Scaffold, we observed that the results of CaP modifications of the bone-contacting surfaces of MSC-Scaffold prototypes carried out during the potentiostatic process (ECD_V=const_) showed much higher repeatability compared to those of the galvanostatic process (ECD_j=const_).

Experimental CaP modification of the MSC-Scaffold pre-prototypes was carried out in two steps. In the first step, the purpose was to determine the most suitable range of conditions for the ECD_V=const_ process. V_ECD_ values from the −9 to −3V range were applied. This stage of investigations showed that the suitable conditions for the ECD_V=const_ process of CaP modification of complex-shaped bone-contacting surfaces of the MSC-Scaffold prototype are strongly influenced by the geometrical features of the scaffold prototype, i.e., by the distance between the spikes. In the case of insufficient room between the MSC-Scaffold’s spikes, the CaP deposits are found between the spikes instead of on their lateral surface. Hence, the P_Sc200_variant of the MSC-Scaffold pre-prototype was excluded from further research. Based on the characterization of the coating’s physiochemical properties—in terms of structural (EDS) and morphological (SEM) properties, and the weight increase of the deposits—the V_ECD_ range from −5.25 to −4.75V was determined to provide the expected CaP modification of the bone-contacting surfaces of the P_Sc350_ variant of the MSC-Scaffold pre-prototype.

In the second step, the influence of AAT pretreatment was examined by applying the previously determined range of V_ECD_ values that achieve the native biomineral Ca/P ratio in coatings on the lateral surface of the MSC-Scaffold pre-prototypes. The investigation procedure applied in the first step was extended to include EDS surface mapping and quantitative analysis of crystalline phases (XRD). The enhancement of the coverage degree of the lateral spike surfaces and the coverage uniformity were ascertained. AAT pretreatment prevents micro-crack formation on the bone-contacting surfaces of the MSC-Scaffold and also affects the increase of the spikes’ lateral surface coverage. The Ca/P ratios of deposits on the lateral spike surfaces in all modified MSC-Scaffold pre-prototypes are consistent with the Ca/P ratios of native osseous CaPs, and plate-like and needle-like CaP crystals appeared on the bone-contacting surface of the MSC-Scaffold pre-prototypes undergoing the AAT pretreatment.

The best overall results for CaP modification of the bone-contacting surfaces of the MSC-Scaffold pre-prototypes were obtained for the V_ECD_ = −5.00 V—the native biomineral Ca/P ratio of deposits (i.e., the closest values to the Ca/P ratio native osseous CaP) was achieved, as well as the highest average mass growth of the coating and the highest coverage degree of spikes’ lateral surface (even in the case of the MSC-Scaffold prototypes without AAT pretreatment). The numerous micro-cracks observed on the MSC-Scaffold pre-prototypes CaP modified at V_ECD_= −5.00V were prevented by applying the AAT pretreatment, finally providing the highest uniformity in comparison to the other V_ECD_ values.

## 5. Conclusions

The effect of CaP ECD deposition on the MSC-Scaffold prototype is conditioned not only by the appropriate choice of electric parameter values of the ECD process but also on the geometrical features (distance between the spikes) of the MSC-Scaffold. Of the two examined design variants, the distance between spike bases of 200 μm (P_Sc200_) and 350 μm (P_Sc350_), the P_Sc200_ MSC-Scaffold variant appeared to be inappropriate to be CaP modified in the ECD_V=const_ process.

Based on the SEM and EDS studies of deposited CaP coatings together with the measurements of the weight increase for the P_Sc350_MSC-Scaffold pre-prototypes, the most suitable V_ECD_ values for the ECD_V=const_process were from −5.25 to −4.75 V and the best results for CaP modification of their bone-contacting surfaces was obtained at V_ECD_ = −5.00 V; the native biomineral Ca/P ratio of coatings was achieved for all the V_ECD_ values used.

ECD combined with AAT pretreatment prevents micro-crack formation on the bone-contacting surfaces of the MSC-Scaffold prototype and increases the spikes’ lateral surface coverage; the best results for the CaP modification of the bone-contacting surfaces was obtained at V_ECD_ = −5.00 V.

Thus, the biomimetic MSC-Scaffold prototype with desired biomineral coating of native Ca/P ratio on bone-contacting surfaces was obtained for a new kind of entirely non-cemented resurfacing arthroplasty endoprostheses.
